# Establishment of a CT-based prediction model for endotracheal tube size in infants aged <1 year

**DOI:** 10.3389/fped.2026.1796799

**Published:** 2026-06-18

**Authors:** Yan Li, Chao Sun, Yanqiu Wu, Xue Tian

**Affiliations:** 1 Department of Anesthesiology, Peking University People’s Hospital, Beijing, China; 2 Department of Radiology, Peking University People’s Hospital, Beijing, China; 3 Department of Medical Information Center, Peking University People’s Hospital, Beijing, China

**Keywords:** chest CT, cricoid cartilage, infants, preterm infants, tracheal intubation

## Abstract

**Objective:**

By quantifying airway parameters on computed tomography (CT) and incorporating clinical factors such as age and prematurity, this study developed an airway prediction model for infants to provide more accurate guidance for endotracheal tube size selection and insertion depth in patients under one year of age.

**Methods:**

A retrospective analysis of data on infants under one year of age who underwent preoperative anesthesia evaluation at Peking University People's Hospital from January 2020 to December 2022. Inclusion criteria: All enrolled infants underwent chest CT examination, with the scanning range covering the level of the cricoid cartilage. The development of cricoid cartilage was associated with laryngeal cartilage maturity. Its size and ossification degree change with age, sex, height and other factors. Therefore, we collected clinical data including the age, sex, gestational age, preterm birth, height and weight at birth and visit which may affect the shortest diameter of the cricoid cartilage in children. Infants with congenital laryngeal malformations, acquired nutritional deficiencies, hormonal abnormalities such as hypothyroidism, neck tumors and a history of maternal smoking during pregnancy were excluded. Accurately measure the shortest inner diameter of the cricoid cartilage and the distance from the cricoid cartilage to the carina on CT images. SPSS 24.0 software was used for simple and multiple linear regression analysis to screen key factors. Corresponding prediction models were established for the shortest cricoid cartilage diameter and the cricoid-to-carina distance, so as to facilitate the individualized selection of tracheal tube sizes and insertion depths in clinical practice.

**Results:**

In this study, age and preterm birth were identified as independent influencing factors for the shortest cricoid cartilage diameter in infants [*P* = 0.008, 0.005(95% CI: 0.001 to 0.008); *P* = 0.022, −1.261 (95% CI: −2.321 to −0.201)]. The established prediction formulas were as follows: the shortest diameter of the cricoid cartilage in full-term infants (mm) = 6.119 + 0.005 × age; The shortest diameter of the cricoid cartilage in preterm infants (mm) = 6.119 + 0.005 × age-1.261. For the distance from the cricoid cartilage to the carina, age was the only significant influencing factor [*P* = 0.003, 35.725(95% CI: 0.016 to 0.073)], and carina formula was derived as follows: distance from the cricoid cartilage to the carina (mm) = 35.725 + 0.044 × age.

**Conclusion:**

Age and preterm status were independently associated with the shortest cricoid cartilage diameter in infants under one year of age, suggesting that preterm infants may require smaller endotracheal tubes than full-term infants of the same age. Age was also associated with the cricoid-to-carina distance. These CT-based findings may support more individualized airway management in infants. However, despite internal bootstrap validation, the model remains limited by the lack of external validation, small sample size, and highly specific ophthalmologic surgical population; therefore, broader validation is required before clinical generalization.

## 1 Introduction

Infants present unique challenges for airway management because their airway anatomy differs from that of older children and adults. The infant airway is funnel-shaped, with the narrowest point located at the level of the cricoid cartilage rather than the glottis (1). In addition, the airway mucosa is fragile and highly vascular, and the laryngeal cartilage is relatively soft (2). As a result, inappropriate endotracheal tube selection may easily lead to complications. An oversized tube may cause mucosal compression, edema, ischemia, or even subglottic stenosis, whereas an undersized tube may result in air leakage, inadequate ventilation, or accidental extubation.

In clinical practice, endotracheal tube size is often estimated using traditional formulas based on age or weight. However, these methods may be inaccurate in infants, especially in those younger than 1 year of age (3), preterm infants (4), and children with abnormal growth patterns. Although chest computed tomography (CT) can directly measure airway diameter and length and has been used in clinical studies (5), it is not routinely performed for preoperative airway assessment because of concerns regarding radiation exposure, cost, and accessibility (6).

With the development of perioperative medicine, individualized airway management has become increasingly important. This study retrospectively analyzed chest CT images of infants, measured the shortest transverse diameter at the level of the cricoid cartilage and the distance from the cricoid cartilage to the carina, identified influencing factors, and developed predictive formulas to provide individualized guidance for clinical intubation practice.

## 2 Materials and methods

### 2.1 General information

This study was approved by the Ethics Committee of Peking University People’ s Hospital (approval number: 2023PHB102-001). A retrospective analysis was conducted on pediatric patients who received preoperative anesthesia evaluation and chest CT examination between January 2020 and December 2022. All enrolled infants were younger than one year, with ASA grade I-III. Exclusion criteria: Incomplete recording of basic information of the patient; chest CT images failing to cover the cricoid cartilage level; congenital laryngeal malformations; acquired nutritional deficiencies; hypothyroidism and other hormonal disorders; neck tumors; as well as a history of maternal smoking during pregnancy.

### 2.2 Data collection

Through a retrospective search of the anesthesia record system at Peking University People's Hospital, the basic information of all pediatric patients from January 2020 to December 2022 was recorded, including chronological age, gender, gestational age, birth weight (low birth weight infants < 2.5 kg; normal birth weight infants 2.5–4 kg), as well as factors such as height and weight during CT examination that may affect the size of the child's cricoid cartilage and the distance from the cricoid cartilage to the carina. The chest CT scans of infants and young children who meet the inclusion criteria were then screened by the imaging system.

**Figure 1:**
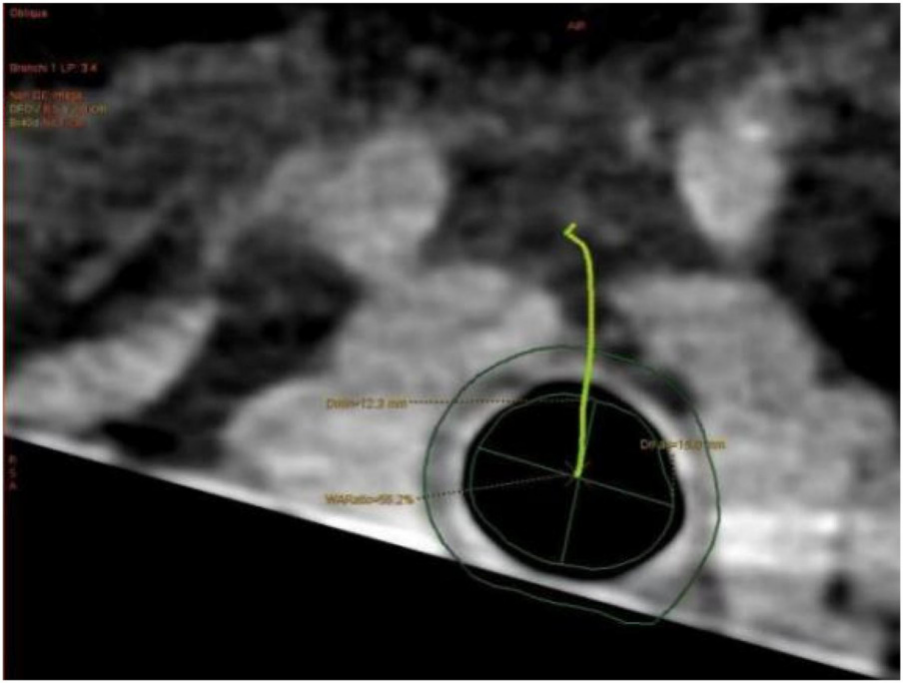
Shortest diameter of the cricoid cartilage observed on chest CT.

**Figure 2:**
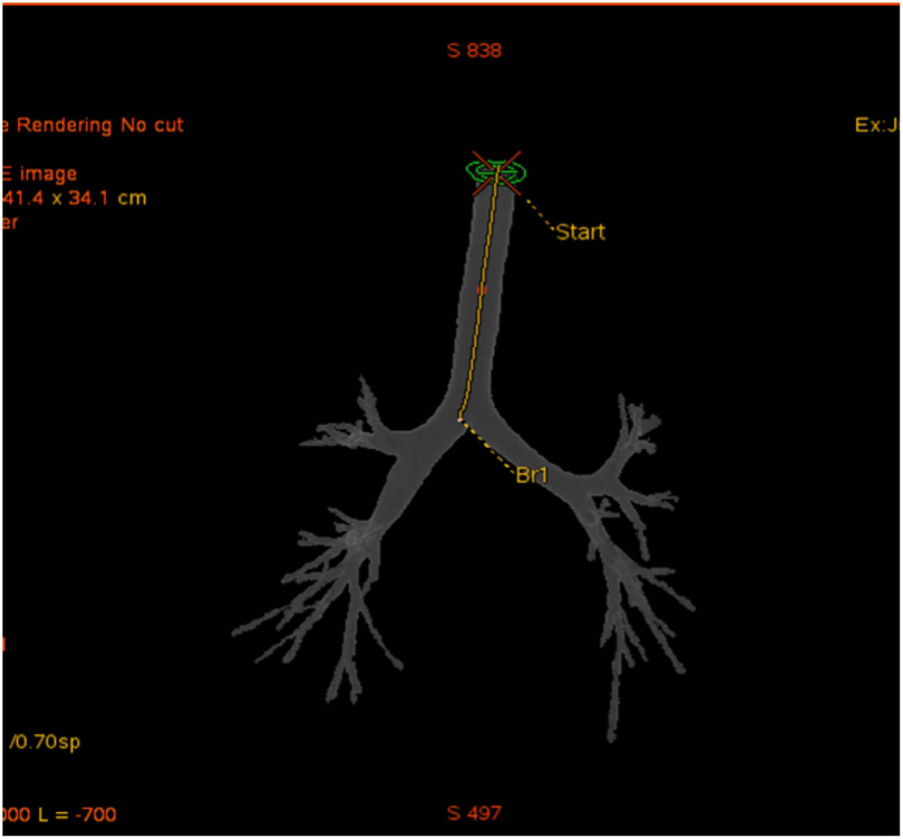
3d mapping measurement of the distance from the cricoid cartilage to the carina.

Key parameters including the shortest diameter of the cricoid cartilage (Figure 1) and the distance from the cricoid cartilage to the carina (Figure 2) were measured based on chest CT images. To ensure the accuracy and reliability of the measurements, all CT measurements were perfomed out by two independently and experienced radiologists, and the average values were adopted. All imaging data were further reviewed by senior radiologists to ensure data validity.

### 2.3 Statistical analysis

This study adopted a retrospective design, and all eligible cases were included in for analysis. Six potential influencing factors were analyzed, including age, sex, gestational age, birth weight, as well as height and weight measured at the time of chest CT examination. Based on the statistical requirement of at least 5 cases are per subgroup, a minimum sample size of 30 pediatric cases was determined for this study. SPSS 24.0 software was used for data processing. Normally distributed measurement data was represented as mean ± standard deviation ($\bar{x}\pm s$), while non-normal distributed measurement data was represented as median and interquartile range [M (IQR)]. Use simple linear regression analysis to identify independent variables that affect the shortest diameter of the cricoid cartilage and the distance from the cricoid cartilage to the carina. Then, the independent variables and dependent variables that have a certain linear relationship (*P* < 0.2) with the dependent variable in simple linear regression analysis are introduced into multiple linear regression analysis to obtain the specific linear relationship between the two. On this basis, a regression formula is established to predict the short diameter of the circular soft bone cricoid cartilage and guide the selection of tracheal tube size. We used the bootstrap technique to generate 50,000 resamples to validate the generated calibration equation and the multivariate prediction model within the equation group, reporting R^2^, RMSE, and prediction intervals. Analyses were performed using SAS Viya for Learners and MedCalc v23.4, with statistical significance set at α=0.05.

## 3 Results

This study included a total of 40 pediatric patients, including 25 males (62.5%) and 15 females (37.5%). Among these children, there were 31 preterm infants (77.5%) and 9 full-term infants (22.5%). The Shapiro Wilk/K-S test in SPSS24.0 (*n* < 50) was found that the distance from the cricoid cartilage to the carina follows a non-normal distribution, whereas all other indicators were normally distributed. The average gestational age at birth was 32 (32 ± 5.1) weeks. The average birth weight was about 1.8 (1.8 ± 0.9) kg. The average age was 196 (196.5 ± 96.1) days. When receiving a chest CT scan, the average height of the patient was 61 (61.6 ± 8.5) cm and the average weight was 8.7 (8.7 ± 2.2) kg. The average shortest diameter of the cricoid cartilage was measured to be 5.8 (5.8 ± 0.8) mm. As the distance from the cricoid cartilage to the carina did not follow a normal distribution, it was presented as median (interquartile range) [M (IQR)], with a value of 42 (17.6) mm. All included children had primary ophthalmic diseases, mainly including retinopathy of prematurity (ROP), familial exudative vitreoretinopathy (FEVR), retinoblastoma (RB), and retinal detachment (RD). Of these patients, 38 had no other comorbidities, 2 had patent foramen ovale, and 1 had mild mitral regurgitation. A total of 36 children received fundus screening and follow-up after treatment, while 5 underwent laser or drug therapy. Relevant baseline data are summarized in Table 1. Setting the shortest diameter of the cricoid cartilage as the dependent variable and other measurement indicators as independent variables, a simple linear regression analysis was conducted. The results showed that the shortest diameter of the cricoid cartilage had a significant linear correlation with age and preterm status (Table 2).

**Table 1:** Basic information of children.

Basic information	Statistics
Sex	
Male (%)	25.0
Female (%)	15.0
Birth gestational age (weeks)	32.0 ± 5.1
Birth weight (kg)	1.8 ± 0.9
Age in days (d)	196.5 ± 96.1
Estimated weight (kg)	8.7 ± 2.2
Estimated length (cm)	61.6 ± 8.5
Shortest horizontal diameter of cricoid cartilage (mm)	5.8 ± 0.8
The distance from the cricoid cartilage to the carina (mm)	42 (17.6)
Disease status (%)	
Retinopathy of prematurity	75.0
Familial exudative vitreoretinopathy	7.5
Retinoblastoma	5.0
Retinal detachment	12.5
Cardiac complication (%)	
No complication	92.5
Patent foramen ovale	5.0
Mild mitral regurgitation	2.5
Surgical status (%)	
Fundus screening	87.5
Eye injection/laser therapy	12.5

**Table 2:** Linear regression analysis of independent variables and the shortest diameter of cricoid cartilage.

Variable	Unstandardized b	SE	Standardized *β*	*t*	*P*-value	95%CI (lower, upper)
Constant	5.857	0.257		22.753	0.000	5.320, 6.394
Male(0)/female (1)	0.027	0.382	0.016	0.072	0.944	−0.769, 0.824
Constant	7.025	0.569		12.339	0.000	5.837, 8.213
Term(0)/preterm (1)	−1.272	0.597	−0.430	−2.129	0.046*	−2.517, −0.026
Constant	5.639	0.514		10.962	0.000	4.554, 6.725
Birthweight stratification	0.102	0.336	0.073	0.304	0.765	−0.607, 0.811
Constant	4.968	0.376		13.204	0.000	4.183, 5.752
Age in days	0.005	0.002	0.511	2.660	0.015*	0.001, 0.009
Constant	4.186	1.845		2.269	0.044	0.126, 8.246
Estimated length	0.026	0.031	0.244	0.834	0.422	−0.042, 0.093
Constant	5.921	0.566		10.463	0.000	4.741, 7.102
Estimated weight	−0.008	0.081	−0.022	−0.098	0.923	−0.176, 0.161

* *P* < 0.2.

Dependent variable: the shortest diameter of cricoid cartilage.

**Figure 3:**
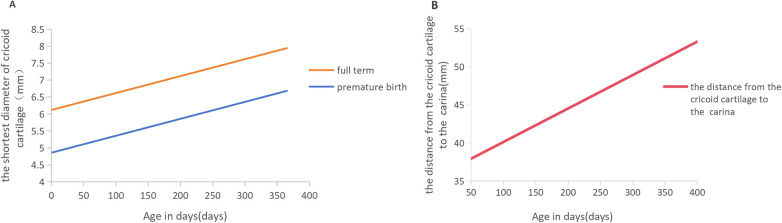
Growth curves of the shortest diameter of the cricoid cartilage and the distance from the cricoid cartilage to the carina in pediatric infants. **(A)** The shortest diameter of the cricoid. For full-term infants, the prediction formula is: y = 6.119 + 0.005x; For preterm infants, the prediction formula is: y = 6.119 + 0.005 × _1_ − 1.261 × _2_. **(B)** The distance from the cricoid cartilage to the carina. The prediction formula is: y = 35.725 + 0.044x.

In the univariate analysis, it was shown that age and preterm status were related to the shortest diameter of the cricoid cartilage (*P* < 0.2). Therefore, the two variables were further included in multiple linear regression analysis, with the shortest diameter of the cricoid cartilage as the dependent variable (Table 3). The results showed that both age and preterm status remained independently correlated with the shortest diameter of the cricoid cartilage (*P* < 0.05). The constant refers to the intercept, indicating the value of the dependent variable when all independent variables were zero; the regression coefficient represents the slope, representing the change in the dependent variable per every increment of the independent variable. Based on the results of multiple regression analysis of the shortest diameter of the cricoid cartilage, the following formula for predicting the short diameter of the cricoid cartilage was established: y = 6.119 + 0.005 × _1_ − 1.261 × _2_ (Figure 3A). In the formula, y represents the shortest diameter of the cricoid cartilage (mm), x_1_ represents age in days, and x_2_ represents preterm status (term x_2_ = 0, preterm x_2_ = 1). Model predictions showed that preterm infants at the same age had an average shortest diameter of the cricoid cartilage that was 1.261 mm smaller than full-term infants, indicating that preterm infants typically require smaller endotracheal tubes. The verification results of bootstrap are detailed in Supplementary Table S1.

**Table 3:** Multiple linear regression analysis of the shortest diameter of cricoid cartilage.

Variable	Unstandardized b	SE	Standardized *β*	*t*	*P*-value	95%CI (lower, upper)
Constance	6.119	0.571		10.713	0.000	4.924, 7.315
Term/preterm	0.005	0.002	0.508	2.968	0.008*	0.001, 0.008
Age in days	−1.261	0.506	−0.426	−2.490	0.022*	−2.321, −0.201

* *P* < 0.05.

In summary, we conducted a simple linear regression analysis using the distance from the cricoid cartilage to the carina as the dependent variable and the other factors as independent variables. The analysis results showed that only age had a linear relationship with tahe distance from the cricoid cartilage to the carina (*P* < 0.05). The detailed results of this analysis were shown in Table 4. A simple linear regression analysis of the relationship between age and the distance from the cricoid cartilage to the carina is detailed in Table 5. Based on the analysis results, the following prediction model was constructed: y = 35.725 + 0.044x (Figure 3B). In the model, y was the airway length (mm) and x was the age in days. The verification results of bootstrap were detailed in Supplementary Table S2.

**Table 4:** Linear regression analysis of the distance from the cricoid cartilage to the carina.

Variable	Unstandardized *b*	SE	Standardized *β*	*t*	*P*-value	95%CI (lower, upper)
Constant	43.720	1.907		22.921	0.000	39.862, 47.578
Male/female	−1.826	3.053	−0.095	−0.598	0.553	−8.002, 4.350
Constant	41.991	2.882		14.568	0.000	36.161, 47.821
Term/preterm	1.389	3.370	0.066	.412	0.682	−5.427, 8.205
Constant	42.836	1.741		24.601	0.000	39.304, 46.367
Birthweight stratification	0.364	3.394	0.018	0.107	0.915	−6.520, 7.248
Constant	35.725	2.671		13.373	0.000	30.321, 41.129
Age in days	0.044	0.014	0.450	3.148	0.003*	0.016, 0.073
Constant	42.686	14.567		2.930	0.008	12.552, 72.821
Estimated length	0.009	0.234	0.008	0.038	0.970	−0.476, 0.494
Constant	37.680	4.578		8.230	0.000	28.420, 46.941
Estimated weight	0.810	0.660	0.193	1.228	0.227	−0.524, 2.144

* *P* < 0.05.

**Table 5:** Linear regression analysis of the relationship between age and the distance from the cricoid cartilage to the carina.

Variable	Unstandardized *b*	SE	Standardized *β*	*t*	*P*-value	95%CI (lower, upper)
Constant	35.725	2.671		13.373	0.000	30.321,41.129
Age in days	0.044	0.014	0.450	3.148	0.003*	0.016,0.073

* *P* < 0.05.

## 4 Discussion

In clinical practice, the selection of endotracheal tube size for children often refers to Cole's formula (internal diameter = age ÷ 4 + 4), which was proposed in 1957. Furthermore, literature indicates that Cole's formula had an error rate of 19% in children aged 2–8 years. Numerous scholars had reviewed the research progress in pediatric endotracheal intubation and summarized multiple formulas for pediatric endotracheal tube sizing. Authors such as Karsli, C (7) had used data to demonstrate the pediatric airways were dynamic and unpredictable, and a previous successful intubation does not guarantee future ease. All pediatric airway management should be performed with high vigilance and adequate preparation. This study emphasizes the importance of individualized, real-time assessment in clinical decision-making, rather than over-reliance on traditional empirical formulas. In particular, abnormal development in preterm infants (8) such as subglottic stenosis and laryngomalacia (9) requires more precise prediction methods, whereas dedicated sizing formulas were currently lacking. Further research focusing on this special population is urgently needed to establish safer criteria for endotracheal tube selection.

CT scanning has important value in the clinical application of bronchial general anesthesia, especially in thoracic surgery. Preoperative chest CT examination can select the appropriate endotracheal tube size for each specific patient, which plays a crucial role in evaluating possible difficult intubation situations, achieving individualized tracheal tube selection for patients, reducing anesthesia complications, and ensuring ventilation function. However, the application of CT-guided endotracheal intubation in infants remains limited, mainly due to the risk of ionizing radiation and the feasibility of clinical implementation. This study was conducted during the COVID-19 pandemic. Pediatric chest CT data were collected under special clinical conditions, offering a valuable opportunity for the present research.

This study used CT to measure the shortest diameter of the cricoid cartilage, aiming to guide the selection of endotracheal tube size (the cricoid shortest diameter determines the maximum allowable diameter for endotracheal intubation). The main reason was that the cricoid cartilage formed a complete and relatively rigid cartilage ring and represented the narrowest part of the airway in infants and young children, making it the best choice for predicting endotracheal tube size (10). Moreover, the measurement technique is simple and convenient. We considered all factors that may affect the shortest diameter of the cricoid cartilage, including age, sex, gestational age, birth length, birth weight, as well as height and weight at the time of CT examination through simple and multiple linear regression analysis, based on these analysis results, age and preterm birth were identified as two independent factors for the shortest diameter cricoid cartilage diameter in infants. According to the regression analysis equation, it can be clearly seen that for infants at the same age, when choosing the type of tracheal intubation, the outer diameter of the catheter in preterm infants should be approximately 1.261 mm smaller than that for full-term infants. Javia et al. tracked 612 cases of intubated preterm infants and found that the incidence of subglottic stenosis was 9.8% (severe cases requiring surgical intervention accounted for 3.2%). This complication was strongly associated with over-sized endotracheal tubes; each 0.5 mm increase in tube outer diameter elevated the risk by 3.4-fold. Therefore, selecting the same endotracheal tube size for preterm infants as that used for term infants may increase the related intubation risk by 8.57-fold. This findings underscore the importance of precise selection. For the distance from the cricoid cartilage to the carina, age was the only significant influencing factor. The measured cricoid-to-carina distance reflects only a partial length of the main trachea and cannot guide the depth of tracheal intubation. It can only be used as a reference indicator for intubation depth. In future work, there may be a sustained need for research on the selection of endotracheal tube size for chest CT examination in infants and special populations, in order to optimize clinical procedures, minimize risks, and improve intubation success rates.

Regarding the difference between the tracheal inner diameter measured by CT scanning and the actual tracheal inner diameter (11), Kuo et al. found that in children under 5 years old, the CT measurement error was larger (± 0.8 mm), especially for airways with a diameter <4 mm. The article also provides a correction formula: actual inner diameter (mm) = 0.92 × CT measurement value −0.3. However, the applicability of this formula is limited. Their study enrolled children aged no younger than 2 years and excluded preterm infants; therefore, the formula is not suitable for the present cohort. In this study, only one patient had a tracheal measurement value of less than 5 mm. To guarantee measurement accuracy, all assessments were performed by senior radiologists, and the average of three repeated measurements was adopted to reduce measurement bias.

The vast majority (92.5%) of the children included in this study underwent surgery and CT examination due to ophthalmic diseases (mainly ROP). ROP children were mostly preterm infants (12), with a very high proportion (77.5%), but usually they had no serious cardiopulmonary complications. Their primary clinical risks are prematurity-related vulnerability, such as susceptibility to intraventricular hemorrhage and infection, rather than structural cardiopulmonary malformations or airway anomalies. Notably, these patients rarely present with congenital anatomical abnormalities including laryngomalacia and tracheal stenosis. The challenge of intubation mainly comes from technical difficulties (such as the selection of appropriate endotracheal tube sizes). Therefore, the condition of these children cannot represent all infants under 1 year of age who require intubation (such as pneumonia, congenital heart disease, abdominal surgical emergencies, trauma patients, etc.). Caution should be exercised when extrapolating the results to a wider range of infant populations, especially non-ophthalmic surgery or critically ill infants. Future validation needs to include a wider range of diseases.

There are still some limitations to this study. Firstly, the small sample size of this study may limit the generalizability and reliability of the research conclusions. Although the model identified preterm birth as a significant factor, the estimation of this coefficient may not be stable enough in cases where there are few full-term cases. Because the overall sample size was relatively small and the majority of children in our cohort were preterm infants, further stratified analysis between full-term and preterm infants was not performed. However, by increasing the sample size, future research can improve the representativeness of research conclusions and may reveal more subtle influencing factors. Secondly, this study is a single center retrospective study, and in the future, the current findings should be confirmed through multi center promotion and external validation to improve the generalizability of the research. Only internal validation using bootstrap resampling was performed in the present study, whereas external validation was not conducted. Therefore, the generalizability and clinical utility of the model still require further confirmation in independent cohorts. Most included patients underwent ophthalmic surgery, which may also limit the generalizability of the findings to other surgical populations. Thirdly, the sample may not fully represent all infants under 1 year old who require endotracheal intubation (such as surgical, critically ill children, etc.), and extrapolation of results should be cautious.

In summary, when choosing the type of endotracheal tube size for infants, consideration should be given to their age after birth and whether they are preterm infants. Preterm infants usually require relatively smaller endotracheal tubes to accommodate narrower airway diameters. Although this factor is acknowledged in our daily work, clinical evidence regarding the exact magnitude of size difference between preterm and term infants remains lacking. Our study provided digital evidence based on imaging measurements for this purpose. These findings had practical application value for optimizing pediatric anesthesia management and reducing airway-related complications. In the future, if the predictive formula for the shortest diameter of cricoid cartilage obtained in this study is validated through prospective multicenter research, it can more accurately guide the selection of endotracheal tube size for infants and young children.

**Figure 4:**
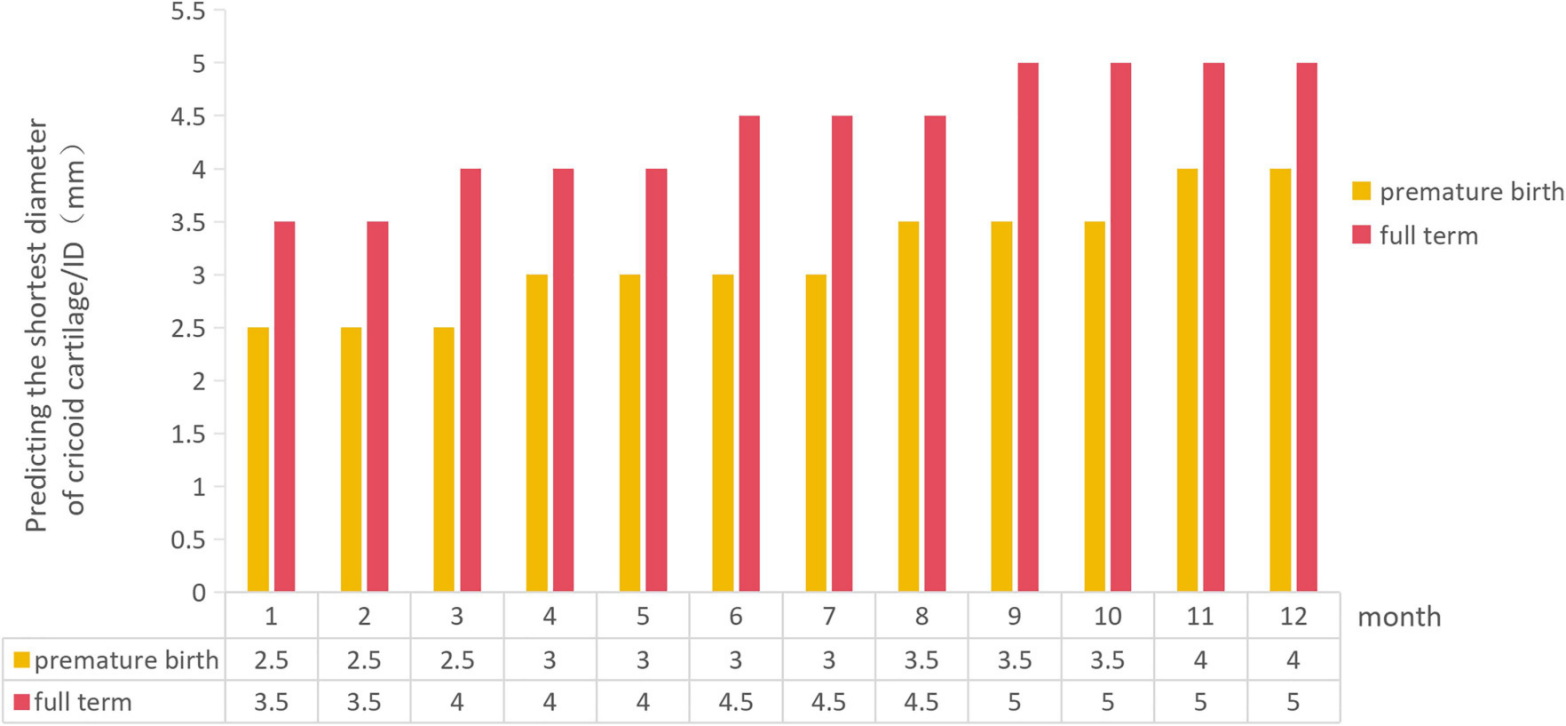
Predicted shortest diameter of cricoid cartilage and optimal tracheal intubation model for children of different month.

Clinical application reference: Taking endotracheal tubes manufactured by Weili Company as an example, the outer diameters of various models were detailed in Table 6 the predicted airway diameters of pediatric patients based on this study were shown in Table 7 and Figure 4. For example, for a 90-day-old full-term infant, the shortest diameter of the cricoid cartilage is 6.569 mm, and an ID 4.0 endotracheal tube is preferred; For preterm infants, the predicted shortest diameter of the cricoid cartilage is 5.308 mm, and an ID 3.0 endotracheal tube is preferable. Nevertheless, the clinician should also prepare the next two adjacent tube sizes in advance according to clinical experience. It should be emphasized that endotracheal tubes produced by different manufacturers may vary in specifications, material, and performance. In clinical practice, each hospital should fully consider the characteristics of the manufacturer of the endotracheal tube used, and carefully choose the appropriate endotracheal tube based on their own clinical experience and individual patient conditions. At the same time, during the entire process of tracheal intubation, medical staff should closely monitor the infant's signs and airway reactions of the child to ensure the safety and effectiveness of the operation, and to facilitate respiratory management and subsequent clinical treatment.

**Table 6:** Outer diameters and cuff filling diameters of different endotracheal tubes (welled, disposable sterile reinforced flexible endotracheal tubes).

Tracheal intubation model (ID)	Outside diameter (mm)	Filling the diameter of the cuff (mm)
1.0	3.3	
1.5	3.8	
2.0	4.3	
2.5	4.8	
3.0	5.3	8.0
3.5	5.8	9.5
4.0	6.3	9.5
4.5	6.8	13.0
5.0	7.3	13.0
5.5	7.8	17.0

**Table 7:** Predicted shortest diameter of cricoid cartilage and optimal tracheal intubation model for children of different ages.

Month of age (month^a^)	Full term		Preterm status	
	Predicting the shortest diameter of cricoid cartilage (mm)	Tracheal intubation model (ID)	Predicting the shortest diameter of cricoid cartilage (mm)	Tracheal intubation model (ID)
1	6.124–6.269	3.5	4.863–5.008	2.5
2	6.274–6.419	3.5	5.013–5.158	2.5
3	6.424–6.569	4.0	5.163–5.308	2.5
4	6.574–6.719	4.0	5.313–5.458	3.0
5	6.724–6.869	4.0	5.463–5.608	3.0
6	6.874–7.019	4.5	5.613–5.758	3.0
7	7.019–7.169	4.5	5.763–5.908	3.0
8	7.174–7.319	4.5	5.913–6.058	3.5
9	7.324–7.469	5.0	6.063–6.208	3.5
10	7.474–7.619	5.0	6.213–6.358	3.5
11	7.624–7.769	5.0	6.363–6.508	4.0
12	7.774–7.919	5.0	6.513–6.658	4.0

a remarks: 1month = 30days.

## Data Availability

The original contributions presented in the study are included in the article/Supplementary Material, further inquiries can be directed to the corresponding author.
