# Supplementary material for: Establishment of a CT-based prediction model for endotracheal tube size in infants aged <1 year
Source: Front Pediatr. 2026 Jun 18;14:1796799. doi: 10.3389/fped.2026.1796799 (PMC13323497; doi:10.3389/fped.2026.1796799)
Supplement: Supplementary file 1 [file Table1.docx]

**Table S1**: Original and bootstrap regression analyses to generate the multivariate prediction equation for the shortest inner diameter of the cricoid cartilage(n =40)

Original

Root mean squared error (RMSE) = 0.635 R2= 0.443 Adjusted R2 = 0.384

| **Variable** | **DF** | **Estimate** | **Standard Error** | **t Value** | **P Value** | **Lower 95% CI** | **Upper 95% CI** |
| --- | --- | --- | --- | --- | --- | --- | --- |
| Intercept | 1 | 6.119 | 0.571 | 10.713 | 0.000 | 4.924 | 7.314 |
| Premature birth or full term | 1 | -1.261 | 0.002 | -2.490 | 0.022 | -2.321 | -0.201 |
| Age in days | 1 | 0.005 | 0.506 | 2.968 | 0.008 | 0.001 | 0.008 |

Bootstrap analysis of 50000 resamples to generate the multivariate prediction equation withthe shortest inner diameter of the cricoid cartilage

| **Variable** | **N** | **Mean** | **Std Dev** | **Lower 95% CI** | **Upper 95% CI** |
| --- | --- | --- | --- | --- | --- |
| Intercept | 50000 | 5.9903 | 0.51546 | 4.60602 | 6.79921 |
| Premature birth  or full term | 50000 | -1.26669 | 0.1793 | -1.61842 | -0.91159 |
| Age in days | 50000 | 0.00482 | 0.00158 | 0.00149 | 0.00769 |
| RMSE | 50000 | 0.59584 | 0.10594 | 0.37624 | 0.79284 |
| R2 | 50000 | 0.45734 | 0.17107 | 0.12118 | 0.78045 |
| Adjusted R2 | 50000 | 0.40483 | 0.18463 | 0.04854 | 0.75752 |

RMSE: Root mean squared error. R2: Coefficient of determination.

**Table S2:** Original and bootstrap regression analyses to generate the multivariate prediction equation for the distance from the cricoid cartilage to carina (n = 40)

Original

Root mean squared error (RMSE) = 8.344 R2= 0.202 Adjusted R2 = 0.182

| **Variable** | **DF** | **Estimate** | **Standard Error** | **t Value** | **P Value** | **Lower 95% CI** | **Upper 95% CI** |
| --- | --- | --- | --- | --- | --- | --- | --- |
| Intercept | 1 | 35.725 | 2.671 | 13.373 | <.0001 | 30.321 | 41.129 |
| Age in days | 1 | 0.044 | 0.014 | 3.148 | 0.003 | 0.016 | 0.073 |

Bootstrap analysis of 50000 resamples to generate the multivariate prediction equation with the distance from the cricoid cartilage to carina

| **Variable** | **N** | **Mean** | **Std Dev** | **Lower 95% CI** | **Upper 95% CI** |
| --- | --- | --- | --- | --- | --- |
| Intercept | 50000 | 35.65302 | 2.56976 | 30.60946 | 40.68569 |
| Age in days | 50000 | 0.04515 | 0.01599 | 0.01466 | 0.07734 |
| RMSE | 50000 | 8.04808 | 1.04173 | 6.0147 | 10.11497 |
| R2 | 50000 | 0.22506 | 0.12149 | 0.02503 | 0.48412 |
| Adjusted R2 | 50000 | 0.20519 | 0.1246 | 0.00004 | 0.47089 |

RMSE: Root mean squared error. R2: Coefficient of determination.
